# Lived Experiences of Iranian Novice Nursing Faculty in Their Professional Roles

**DOI:** 10.5539/gjhs.v7n6p138

**Published:** 2015-04-03

**Authors:** Abbas Heydari, Seyed Masoud Hosseini, Hossein Karimi Moonaghi

**Affiliations:** 1 Evidence-Based Caring Research Center, Department of Medical-Surgical Nursing, School of Nursing and Midwifery, Mashhad University of Medical Sciences, Mashhad, Iran; 2 School of Nursing and Midwifery, Mashhad University of Medical Sciences, Mashhad, Iran

**Keywords:** nursing faculty, qualitative research, needs assessment

## Abstract

Many studies have focused on understanding the novice nursing faculty experiences on their roles and challenges that they encounter. But there are merely little evidences about Iranian novice nursing faculty experiences to perform their roles. In many universities of Iran, a novice faculty has to attend in a set of educational workshops (e.g. teaching and assessment methods), which are not based on their specific needs. Qualitative approach may provide first hand data needed to understand novice nursing faculty perceptions to develop an actual empowerment program. A qualitative design based on phenomenological approach was applied to uncover the meaning of novice nursing faculties’ lived experiences in Mashhad University of Medical Sciences (Iran). Five main themes emerged from data as: “Importance of support”, “Bewilderment”, “Efficiency concern”, “Concern of being accepted” and “Clinical education: Walking on the edge”. These themes implied to uncertainty about fulfilling the academia expectations by novice faculty, desire to overcome the challenges, time limitation, and, unfriendly behavior from senior colleagues. Findings support that more than formal programs, such as workshops, the novice faculty can be empowered by other approaches (e.g. mentorship programs) where they can safely transit into academia under supervision of experienced colleagues. Also, this may facilitate the socialization process of novice faculty to academia and clinical fields.

## 1. Introduction

The main mission of nursing schools is to train competent and qualified nurses with sufficient knowledge, attitude and skills to protect and improve public health (AACN, 2006; Shipman & Hooten, 2008). Nurse educators as students’ advisors, perform a decisive role in learning process (Allen, 2008; Jackson et al., 2008). Experienced faculty shortage can have significant effects on nursing students training process, leading to unqualified nurses for the future (Potempa, Redman, & Landstrom, 2009; Reid, Hinderer, Jarosinski, Mister & Seldomridge, 2013). Due to fast growth of higher education in the past decades, providing qualified faculty is still a challenge for educational administrators in Iran and many countries (Gholami & Asady, 2014; WHO, 2006). Although, there is no valid data on demographic and career features of nursing faculty in Iran, it seems that retirement issue and aging of qualified nurse educators do not differ from other countries. Recruitment of qualified nurse educators to replace retired ones has always been a concern for nursing administrators (Allen, 2008; Potempa et al., 2009; Reid et al., 2013; Tanner, 2005). Employing young teachers to undertake the task of training, makes it possible to benefit from their energy and potentials for a longer time (Halstead, 2012). However, current changes in nursing education have led to particular needs, especially for the novice teachers (Clark, Houten, & Perea-Ryan, 2010; Link & Scholtz, 2000; Neely-Smith, 2008; Skiba, 2007). Nurse educators are expected to play multiple roles competently (NLN, 2005). The expectation to play different roles by novice nurse educators, results in tensions and stress for them (Boyden, 2000; Dhed & Mollica, 2013; Gazza, 2009; Weidman, 2013).

Better perception of novice teachers’ experiences in their professional roles is essential to design more efficient empowerment programs for them (Duphily, 2011). Educational workshops (e.g. teaching and assessment methods) and brief orientation sessions are common approaches in many universities to empower novice faculty, but they are not usually based on their actual and specific needs. Although, many reports have emphasized on benefits of mentorship programs to prepare novice faculty for their roles (Dhed & Mollica, 2013; Heinrich & Oberleitner, 2012; Peters & Boylston, 2006; Sculley, Myrick & Paul, 2013); Reid et al have reported that there is no agreement on methods and duration of orientation programs for novice teachers (Reid et al., 2013). The important point is to plan educational programs based on needs assessment research findings, and must be individualized to be more efficient (Dhed & Mollica, 2013).

Many studies have been conducted concerning the subject of the current research. In one study has been reported that “adapting with academic social environment” was the most important stressor among novice nurse educators (Sawatzky & Enns, 2009). Hessler and Ritchie in their study provided ten suggestions to facilitate hiring and retaining of novice nurse educators. Many of their recommendations have focused on providing guidance, facilitating the socialization process, flexibility and patience to the novice teachers (Hessler & Ritchie, 2006). In a study to describe and interpret the novice faculty experiences in their transition from clinical nurse expert to novice educator, author concluded that nursing program administrators play an important role to facilitate this transition by providing additional support for clinical nurse experts in educational theory and evaluation skills areas (Weidman, 2013). Some studies have emphasized on implementation of a formal mentoring program for novice faculty (Cangelosi, 2014; Schoening, 2013). In these studies, many of the participants had felt a lack of formal mentoring and training program. Although some of them had formal education in teaching and learning practices, yet they expressed need to be guided by senior faculty. Cangelosi mentioned that the schools of nursing must provide enough time and resources for senior faculty to formally guide novice ones (Cangelosi, 2014). As mentioned earlier, the important point is that empowering plan for novice faculty must be tailored based on actual needs of targeted audiences to be more effective (Billings & Halstead, 2013; Dhed & Mollica, 2013; Grant, 2002). The purpose of this study was to perceive lived experiences of Iranian novice nursing faculty in their professional roles, and providing recommendations for nursing educational administrators to plan novice faculty empowering programs more efficient.

## 2. Method

A phenomenological approach was applied in this study. The purpose of phenomenology is to perceive nature and meaning of lived experiences in daily life of individuals (Patton, 2002). In phenomenology, it is attempted to clarify structure or essences of a person’s lived experiences related to a phenomenon; it looks for unity in content and meaning, that leads to identifying of phenomenon and appropriately interpreting it via daily lived experiences (Streubert & Carpenter, 2010).

The current study endeavors to explain the meaning of “novice nursing faculty” experiences. The study population was faculty members of selected schools of nursing from Razavi, Northern, and Southern Khorasan provinces at the northern east of Iran. The participants were enrolled into the study via a purposeful sampling and snowball method. First, the eligible faculty members were invited to participate. Then, they were asked to introduce their colleagues with similar condition. The inclusion criteria of the participants were as follows: he/she should be a faculty member of nursing school, with less than 3 years of work experience and willing to take part. Firstly, deans of nursing schools helped to recognize the population and then the eligible ones were contacted to set an appointment for interview in the Mashhad Nursing and Midwifery School or in their local schools.

## 2.1 Participants

Participants were nine faculty members (Seven females and two males) from four nursing schools with mean age of 31.33±2.74 years who had the study inclusion criteria. Of the participants, two had PhD and the rest had M.S. degree in nursing with mean of 2.19±0.84 years of work experience.

## 2.2 Data Collection

Data were gathered by in depth unstructured interviews and field notes since May 2013 through April 2014. Each interview lasted about 30 to 70 minutes. MP3 recorder device was used to record the interview sessions. Participants were asked to describe one of their memorable experiences in first weeks of their career as a faculty in classroom or clinical fields. To deepen the perception of their experiences, they were asked to clarify that experience more by probing questions. Questions of the interview were as follow:

- Explain an experience that you never forgot it in first weeks of your career as a faculty?
- What were your pleasant and unpleasant experiences in academia in first weeks as a faculty?
- Why did you have such a feeling in that specific situation?
- Can you explain more about it?

Second interview or call was done to complete the data.

## 2.3 Considerations of Ethics

Research Ethics Committee of Mashhad University of Medical Sciences approved the study (Reg. No. 920074). Participation was optional. Before interview, participants were assured of confidentiality of their identity and data. Also, participants’ signed written consent and their contact details were obtained.

## 2.3 Data Analysis

Each recorded interview was transcribed to a text and then coded using MAXQDA software to manage the data. van Manen method was used to analyze the data. van Manen is mostly well-known as a descriptive and interpretive (Hybrid) phenomenologist (Dowling, 2007; Nikbakht, Borimnejad & Joolaee, 2009). He believes that all phenomenological enquiries begin with discourse. A distinctive characteristic of hybrid phenomenology is the perspective about bracketing. Dowling (2007, P.138) quotes from van Manen: “If we simply try to forget or ignore what we already “know”, we might find that the presuppositions persistently creep back into our reflections” (Dowling, 2007). van Mannen described his method as a process consisting of six steps as follow: “1- Turning to the nature of lived experience, 2- Investigating the experience as we live it, 3- Hermeneutic phenomenological reflection, 4- Hermeneutic phenomenological writing, 5- Maintaining a strong and oriented position, 6- Balancing the research context: Parts and whole”(van Manen, 1990).

From the beginning, the idea of this research was shaped based on the first meaning of van Manen method. Also, participant selection was according to second methodological meaning to gather data from the people who have experienced the phenomenon. Data analysis began after the first interview. The interview text was read carefully several times. Sentences and phrases that were related to the purpose of the study were extracted and coded. Based on van Manen method, going back and forth between parts and whole was done by the researchers during the study.

Confirmability and credibility criteria were used; confirmability means that the data should be acceptable and real, which can be improved through allocating sufficient time, using good communication skills, triangulation, peer debriefing, and member check. It was attempted to guarantee the objectivity of results by careful design and implementation of research steps and clear methodology. In order to increase credibility of data, enough time was allocated to gather the data and analyze them in depth. Researchers made a confidential atmosphere so that participants were encouraged to share more experiences. Moreover, members reviewed the study in order to increase credibility. Final results were sent back to participants by email to specify whether the descriptions have reflected their experiences or not. Two qualitative research experts were asked to analyze primary data again (integration of researchers). To reach maximal variation in sample, data were gathered from nursing faculty with different field experiences, degrees and gender. Moreover, dependability and result verification were gained through integrating notes, primary data, extracted meaning and formulated field notes as a whole.

### 3. Results

Finally, after several reviewing, integrating and reducing primary codes, main themes and subthemes of participants’ experiences on “being novice nursing faculty” were extracted as presented in Table 1.

**Table 1:** Themes and sub-themes extracted from participants’ experiences

Raw	Main Themes	Subthemes
1	The importance of support	Need for support
		Search for support
2	Efficiency Concern	Need to be ready for the role
		Gaps in knowledge and skills
3	Bewilderment	Interactions with colleagues
		Dealing with norms and dominant culture in the environment of school and clinic
		Dealing with the School expectations
4	Concern of being accepted	Lack of authority
		Being ignored
		Fearing to be judged unfairly by other
		Lack of respect from clinical staff
		Search for intimacy
5	Clinical training: walking on the edge	Unpredictable situation
		Fighting without weapon

### 3.1 The importance of Support

Participants mostly insisted on the importance of support for better performance both by experienced colleagues and the school administrators. Most of them stated inadequacy of teaching skills courses and insisted on the importance of gaining experience in practice with the support of experienced colleagues. In their opinion, the opportunity to practice teaching beside experienced teachers could help them to use their previous trainings and gain new points about dealing with job requirements which was not possible during study period for M.S. and PhD degree. For instance, a participant said about her experience of support:

“… We have teaching and assessing courses in B.S. and M.S. curricula. Also, during PhD, we study and review new methods of teaching and assessment … at the moment, one thinks that he/she knows everything, while when one enters the real situation, it is very different ….”.

Another participant said:

“…Whatever we have learned is theoretical. We were never thought how to deal with the students in clinic… how to teach … you know that clinical teaching is very difficult, especially for a major like nursing. I mean that no matter how much knowledge one has, he/she will not be beneficial if he/she doesn’t have enough clinical education experience… I do feel the essence of this… it is necessary.”

The other important point is lack of help and support from school administrative system. Mostly, participants declared that the school does not support when needed. They believed that schools’ officials merely pay attention to quantitative aspect of doing tasks, and they do not value the quality improvement. They do not allocate enough time for novice teacher preparation and also do not consider abilities and experiences of individuals in assigning theoretical and clinical courses. These matters lead to lack of support sense among teachers. One participant said:

“When you begin your job, on one side you have to teach theoretical courses, and on the other side, guide students in clinical settings. You do not have time to review your knowledge. You should only think to prepare your courses…I mean if you are employed as a faculty, shouldn’t you be informed to study your courses beforehand? Anyway, we, as a novice teacher are different from experienced ones and should study before class at least for one or two hours, prepare slides and find new things; I don’t have time in practice.”

### 3.2 Efficiency Concern

The other main theme is “efficiency concern” which is much related to the first theme “importance of support”. Most of the participants were to some extent worried about not being efficient to perform their expected role, which increases their stress. A female participant said:

“One of negative points in the class is that I talk too fast, because I simply want to cover the entire course content, and then I have extra time. For example, I plan the class for one hour and forty five minutes and finish after one hour and a quarter … I think that the students do not have time to think! It seems I have too much stress to finish the session and how to present it”.

This may be due to unfamiliarity with new approaches in teaching. Currently, the role of teachers in classes has changed and it seems that they are asked to play as a facilitator for students, not delivering entire of the contents to the students.

Lack of enough opportunity to adopt the roles and new situation is another main concern of novice nursing faculty. On one hand, the novice teacher should answer students and their needs, lead them to educational goals, and from the other hand, should manage extreme expectations of the school. Most of them talked about too much work and lack of time to meet expectations. This matter has influenced personal life of the participants. For example a female married participant said:

“In the first semester, I didn’t know how my child is growing. I mean I had classes till 5 or 6 pm and arrived at home at 7 pm. Then I had to make dinner and again study. Most of the time I couldn’t make dinner. My husband helped me a lot … I always thought that I’m neither a good mother, nor a good wife or a good daughter for my parents. I always thought so. And most importantly, I’m not a good teacher for my students. I thought I’m nothing, I’m too confused. Maybe if I was not too much under pressure, I could feel better and get rid of negative feelings. Then I could make a balance to be a good researcher and teacher, as well as, a good mother and wife.”

These experiences could lead the novice faculty to feel uncertainty about their competencies and induce additional stress to them.

### 3.3 Bewilderment

Moreover, “bewilderment” is another important theme in this study. It has three subcategories including: “Interactions with colleagues”, “Dealing with norms and dominant culture in the environment of school and clinic”, “Dealing with the School expectations”. Most of the participants of the study said that school expectations had not been clear. They said authorities did not set their expectations clearly, which led to conflict among teachers. For instance, a male participant said:

“We try to dance according to any music they play”.

This is a Persian metaphor. This metaphor’s hidden message is adaptation with authorities’ expectations though they are against one’s willingness, to reduce tensions and stresses. Moreover, a subcategory for this theme is bewilderment in dealing with norms and dominant culture in the environment of school and clinic. In most cases, the novice faculty has conflict with dominant culture and norms exist in interactions among novice and experienced teachers. A participant, for example, said:

“I am confused that should I be myself, and ask about ambiguities, faults, questions respectfully, or should I follow the norms of the faculty like others? One of my problems in first semester was worrying about any arising conflicts between I and important members of school… Then I realized about the atmosphere of the school, and got along with them… I finally learned how to manage it.”

### 3.4 Concern of Being Accepted

The other theme related to group and organizational norms is “concern of being accepted”. This theme and its subthemes present the concern of novice faculty that whether he/she would be accepted among colleagues and clinical staff as a capable person or not. In many cases, participants mentioned incivility from senior colleagues and clinical staffs. Part of this feeling might be due to experienced teachers’ concern to lose their position by novice teachers. A participant said:

“Many of experienced teachers want to put down novice teachers. I’m not sure … May be a feeling to lose their status. And I think this attitude is not just … Since their position is fixed… this is the only thing I can say”.

The other one said:

“I mean sometimes I think if I would be more competent in some issues like working with internet, computer and virtual education… I might occupy their position and they lose their status… I really felt this either in Mashhad and there… I think the person we are talking about had the exact feeling”.

The sense of not being accepted by clinical staff might be due to organizational detachment among nursing education centers (School) and clinical services sections (Hospitals). This detachment has led to dissociation of clinical staff from being nursing faculty in Iran; therefore, this group has a negative attitude toward nursing faculty, specially the novice ones. They maybe think that they themselves could be a capable nursing faculty. For instance, a participant mentioned:

“I realized that the behavior of my old friends as clinical staff was changed since my promotion. In the past, I expected they support me, but not now; I have accepted it… it was all stress”.

### 3.5 Clinical Training: Walking on Edges

A challenging arena for the novice teachers is clinical training. Most of the participants mentioned that clinical training lead to considerable stress. It might be due to unpredictability of clinical situations that happens during training for teachers and students.

“Working in clinic is very different from teaching at university. Well, if you are going to work there, so there must be a program to empower you, while there isn’t. Of course, we have different methods for learning, one of them is trial and error, which is costly and time consuming. We learned in this manner.”

The need for more preparation to teach at clinical settings was a point that most of participants mentioned. Having M.S. or PhD degree cannot guarantee one’s efficiency in clinical training. One of participants said:

“Since we do not have clinical experience and go from university to clinical education fields directly, we are not as competent as clinical staffs. They know a lot and I’m not familiar with their routine. I have the knowledge but don’t know what is needed for the ward.”

Another nurse educator said:

“It is in clinical setting that we really face problems, for example, you have prepared yourself for cardiovascular training in ward, while there is a complicated patient with other diseases,…and the students may ask questions… it is too difficult to work in clinical settings”

Totally, the emerged themes in this study indicate that there are many problems for novice nurse educators to perform their expected roles efficiently. But the problems related to teaching competencies, especially in clinical arena, are more considerable. Also participants in this study had a lot of stress related to be accepted in the group.

## 4. Discussion

The emerged themes in this study were based on novice teachers’ needs and actual problems. Grant insists on doing needs assessment for each career and perceiving expressed needs (Grant, 2002). The key point is to allocate sufficient time so that teachers can gain experiences and adapt their profession. Shortage of time and too much work do not let them to adapt appropriately and gain enough experience. Other studies have mentioned similar problem (Gazza, 2009; Sawatzky & Enns, 2009; Schoening, 2013; Siler & Kleiner, 2001; Weidman, 2013).

Many participants insisted on the importance of practical experiences. This may be more crucial in our study because most of the participants had no or little experiences in clinical practice before being hired as a nursing faculty. It is different from many studies that mostly conducted in U.S. and other countries. In those studies, the participants were novice educator with high experiences in clinical practice (Cangelosi, 2014; Reid et al., 2013; Schoening, 2013). Some researchers have emphasized that the nursing schools administrators should provide educational environment and oppurtunities for novice faculty to transit into new roles easier. They agree that having rich clinical experiences by the clinical experts do not necessarily mean that they can teach in academia proficiently (Cangelosi, 2014; Duphily, 2011; Weidman, 2013). Findings of this study showed a gap between knowledge and skills of novice teachers to perform their roles and also, a gap between expectations and reality. Schriner(2007) reported that “loss of preparation for the role”, “clinical training realities” and “not being prepared for teaching role” might cause doubt and uncertainty in novice teachers and reduces their self-confidence (Schriner, 2007).Nursing schools administrators should consider novice faculty developmental needs. Members of any profession need a wide range of knowledge and skills that were not clear for them at the training period. The educational programming should be based on learning needs that have been experienced by individuals themselves, and what the experienced scholars perceive regarding learners’ needs (Grant, 2002).

Preparing M.S. students for education role and delivering needed courses are necessary in M.S. curricula. Recently, there have been revisions in nursing M.S. programs in Iran, and some of the M.S. students are trained to serve as clinical practitioners. Lack of teaching courses will lead to lack of preparation for teaching. However, due to shortage of nurse educators, some of these graduates are hired as nursing faculty. The results of the current study showed that being nursing faculty immediately after M.S. or PhD graduation makes a lot of challenges for teachers and nursing schools, since these novice teachers require to gain more experiences in practice. On the other hand, there are ambiguities and doubts about using clinical experienced individuals in teaching roles, since many of them have not studied teaching courses during M.S. program and need to be prepared. Also, they have worked for several years and are about to be retired. Halstead states that it is advantageous to use young teachers in nursing education (Halstead, 2012). It seems the suitable solution is to incorporate the energy and enthusiasm of novice faculty and experiences of senior ones, in long-term empowerment programs to share educational experiences and to build a relationship between these two groups to facilitate novice faculty transitioning process. Also, the rich experiences of senior faculty can be acknowledged. The results of the current study can be the basis for such programs.

Findings of the study support that there are many challenges for nursing educational administrators in Iran. Due to the universities employment rules in Iran, there is an age limitation for hiring new faculty that do not allow to recruit clinical experienced staff as a faculty. Also, many M.S. degree programs in nursing have focused more to train nurse practitioners than nurse educators. Although, due to faculty shortage and replacing retired ones, many of these graduates are employed as teachers with little or even no preparation for their roles especially in newly established schools. More attention is required to fulfill the novice nurse educators’ needs, especially those who have not trained for a teaching role, and with no or little experiences on clinical education.
